# Trajectories of Parental Warmth and the Role They Play in Explaining Adolescent Prosocial Behavior

**DOI:** 10.1007/s10964-023-01887-3

**Published:** 2023-10-21

**Authors:** Lisa Buckley, Tiffany Atkins, Withanage Perera, Michael Waller

**Affiliations:** The School of Public Health, The University of Queensland, Herston, 4006 QLD Australia

**Keywords:** Prosocial, Parent, Parental Warmth, Trajectory, Adolescent

## Abstract

Adolescent prosocial behavior suggests social competence and it is associated with greater parental warmth yet the experience of warmth through child and adolescent development is not well understood as it relates to such prosocial behavior. A nationally representative dataset from the Longitudinal Study of Australian Children cohort was used. The analyses involved multiple waves beginning when children were aged 4–5. The main analyses used a sample of 2723 adolescents aged 16–17 years (Mean, S.D. = 16.45, 0.50; 49.2% female, 50.8% male). Parental warmth trajectories (from ages 4–5 through 16–17 years) were created and used to explore the accumulated effect of a lifecourse of parental warmth on adolescent prosocial behavior as measured when adolescents were aged 16–17 years. There were three trajectories described as, consistent (28.7%), slight decline (51.4%), and declining warmth (19.8%). These were associated with prosocial behavior; adolescents with a slight decline in warmth were 2.2 times less likely than those with consistent warmth to have the highest prosocial behavior. Consistent parental warmth likely provides greatest benefit for increased prosocial behavior in mid-adolescence.

## Introduction

Adolescent prosocial behavior is desired and reflects social competence with previous research suggesting it is associated with greater parental warmth (Padilla-Walker et al., 2016). Trajectories of parental warmth through child and adolescent development, however, are rarely considered. Where findings do explore the developmental experience, they suggest there is a dip in warmth about the time of puberty (Lansford et al., 2021). Such studies largely test a homogenous pattern of warmth however there may be different trajectories or a heterogenous pattern (Trentacosta et al., 2011). Youth who can be described by such different trajectories potentially may then engage differently in prosocial behavior. The current study tests whether different latent trajectories of warmth from childhood through adolescence are associated with later adolescent prosocial behavior.

Adolescents who report more prosocial behavior typically have many other positive experiences, including better academic performance (Gerbino et al., 2018), better physical health (Schreier et al., 2013) and mental health (Haroz et al., 2013), more engagement in sports (Moejies et al., 2019), as well as civic engagement (Taylor et al., 2018). Providing fewer prosocial behaviors is typically associated with more externalizing behaviors and internalizing problems (Memmott-Elison et al., 2020). Prosocial behavior forms an important part of adolescent interpersonal experiences, and potentially extends those benefits in adolescents’ health, education, and wider social development.

Prosocial behavior is intended and voluntary behavior that supports the well-being of others in a positive and socially responsible manner (Eisenberg et al., 2006). Such behaviors among adolescents may include lending a helping hand, sharing belongings or resources, and providing comfort to someone who is hurt or upset; with these behaviors reflected in the tools typically used to capture prosocial behavior (Corell-García et al., 2019). For example, the Strengths and Difficulties subscale (see Goodman, 2001), conceptualizes prosocial behavior around being considerate of others’ feelings, sharing, acts of kindness, volunteering, and helpful actions.

### Role of Parental Warmth in Prosocial Behavior

The parent-child bond is perhaps one of the more fundamental building blocks for socio-emotional development (e.g., Bowlby, 1979). Parental warmth reflects the attitudes of parents and their conduct in supporting their child with acceptance, affection, and love (Elsaesser et al., 2017). It can be considered a positive regard that is expressed to their child perhaps with pleasant interactions and positive involvement in the child’s activities. Involvement may be coupled with expressions of interest and praise or enthusiasm for accomplishments (Elsaesser et al., 2017).

Parental warmth is associated with attachment (Doyle & Markiewicz, 2005). A secure attachment may foster the resources (internal and external) for the child to manage their emotions and social situations. Being able to manage emotions and self-regulate may enable an adolescent to develop an awareness of, and identify cues in others’ states and their experiences. Parental warmth might allow the development of capacity or internal working models to moderate emotions when attending to others (Williams & Berthelsen, 2017) and more broadly allow children the capacity to be outward focused, to consider others are deserving of support and be able to appreciate the emotions or distress of others. Indeed, children who better able to understand their peer’s emotions typically have a more secure parental attachment (e.g., Laible & Thompson, 2002).

Warmth might also facilitate emotional security and may promote a confidence or ability to not just notice, but also be able to address, the needs and emotions of others. Attachment or the feeling of security enables a willingness to help others (Cassidy et al., 2018). Youth may thus have a sense of security that enables a feeling of confidence to manage their own emotions while attending to others. Parents who provide warmth and support are likely to have children who thus can manage their own socio-emotional processes (attending to own and others’ emotions) but also, in turn, respond to parent’s efforts to direct and guide positive and prosocial interactions. In other words, it also enables the adolescent to be more aware of, and able to internalize, their parents’ messages for more desirable behavior (Eisenberg et al., 2005).

Parents who display limited warmth may however engender socio-emotional dysregulation and greater withdrawal from social situations, such that it then becomes a more common practice to stand back and avoid potential costs of prosocial behavior. Indeed, biobehavioral research further supports the positive role of parents with parental practices mediating the relationship between socio-economic disadvantage and adolescents’ neurological development (Whittle et al., 2017). Adolescents may thus benefit from early parental warmth but it would be important to understand if, and how, this changes with development. For example, whether there are different trajectories of parental warmth that might be typical through childhood and into adolescence and similarly explain socio-emotional skills in adolescence, such as prosocial behavior.

### Parental Warmth Through Childhood and Into Adolescence

Having early parental warmth may allow a foundation for socio-emotional skills that continue to develop over time. Having such individual resources may provide a positive behavioral repertoire that is then consistently reinforced, with a corresponding repetition of positive behaviors. Theoretically, the work of Bandura (1969) would also suggest that if parents continue to exhibit warmth, this would provide models of caring and likely helpful behavior. Such models may stimulate a similar behavior by their children who see prosocial acts by their parents as well as receiving reinforcement (i.e., regular praise) for their own prosocial behavior. Having greater parental warmth over time may enable both the motivation (through having capacity to identify others’ emotions and needs) and the scaffolding (through positive modeling and reinforcement) to enact prosocial behaviors. Parental warmth thus may impact and promote prosocial behavior through several mechanisms that might be particularly strong if parental warmth is consistent through development, that is these potential mechanisms have the opportunity to compound or cumulatively impact on development.

Longitudinal research has shown that warmth provided and experienced in early adolescence predicts prosocial behavior in mid-adolescence (e.g., Kanacri et al., 2021). In an exploration across eleven cultural groups findings suggested a positive association between parental warmth and prosocial behavior during late childhood (Pastorelli et al., 2021). That is, more parental warmth at age 9–10 years was associated with prosocial behavior at 11–12 years (Pastorelli et al., 2021).

Many of the studies focus on a relationship between warmth and prosocial behavior that occurs in late childhood (e.g., Carlo et al., 2010) and early adolescence (e.g., Kanacri et al., 2021) however prosocial behavior continues to be important and relevant through mid-adolescence (Padilla-Walker et al., 2018) at a time when adolescents are increasingly forming new relationships outside of the family and have increasing independence and responsibility for their interactions. They also have more opportunities for prosocial behavior outside the home and are beginning to form more intimate relationships with peers. Parental warmth has been associated with prosocial behavior at age 17 years (Richards et al., 2015) and parental warmth at aged 12 has been associated with prosocial behavior at age 18 (Padilla-Walker et al., 2018). Findings suggest relevance in understanding the role of parental warmth on prosocial behavior into mid- to late- adolescence, reflecting an age where there is continued expansion of relationships and increasingly diverse social interactions.

Few studies have examined the experience of parental warmth through the school years, from childhood through to adolescence (i.e., ages of 4–5 through to 14–15 years). In one exception examining children aged 8–15 years, there was a general decline in parental warmth through puberty across the sample (Lansford et al., 2021). Parental warmth has been shown to stabilize through childhood and similarly has shown a slight decline with the transition to adolescence (Shanahan et al., 2007). It is perhaps not surprising to see a decline in parental warmth through development where this has been studied. Parents may find it challenging to continue to provide the same warmth experience when there may be less time spent and more instances of parent-child conflict typical through adolescence (Branje, 2018). It is not known however how if such a decline is likely similar for all youth and thus can similarly contribute to the likelihood of adolescent prosocial behavior.

There are a few studies suggesting that parental practices may not show a consistent pattern for all adolescents (e.g., Trentacosta et al., 2011). Importantly in explaining prosocial behavior an inconsistent trajectory of warmth might limit some opportunity for continued observation or modeling as well as reinforcement of their own positive behavior. A latent person-centered (rather than variable-centered) approach might help identify individuals who share a similar or approximated profile in their trajectory of parental warmth. The person-centered approach is predicated on an assumption that there are categories of individuals who share a pattern of behavior that is similar to others who share that category and different to those who do not share their category (Laursen & Hoff, 2006) and that there is thus latent heterogeneity in the developmental trajectory of parental warmth through childhood and adolescence. Studies have not examined the trajectories of warmth except in the mother-son relationship (i.e., Trentacosta et al., 2011). In this study they found three trajectories of the sons’ experience; a high consistent maternal warmth, a slight decline over development, and a larger decline over development (Trentacosta et al., 2011). However, this study only looked at the mother-son relationship and did not link such trajectories with adolescent prosocial behaviors and in particular assess if any common trajectories in parental warmth are associated with prosocial behavior.

## Current Study

The aim of the current study is to examine how unique trajectories of parental warmth throughout childhood and adolescence relates to prosocial behavior reported by adolescents at aged 16–17 years, in the last few years of their schooling. Data from the Longitudinal Study of Australian Children (see, Mohal et al., 2021) was used to provide information about warmth across development and on later prosocial behavior. The main hypothesis of the study is that the likelihood of adolescents’ prosocial behavior (at age 16–17 years) will be predicted by unique latent developmental trajectories of parental warmth after controlling for covariates (socio-economic position, gender, family structure, language spoken at home).

## Methods

### Participants and Design

Data was from the nationally representative Longitudinal Study of Australian Children (Mohal et al., 2021). LSAC is a population-based study involving two different cohorts of Australian children. The kindergarten (K) cohort was analyzed in the current study where children were 4–5 years of age at wave 1 in 2004 and followed up approximately every two years where they are age 16–17 years in the final wave used in the current study (wave 7 in 2016).

Sampling for all LSAC data used a two-stage clustered random sampling design beginning with data from Australia’s healthcare database, Medicare (Mohal et al., 2021). Sampling for LSAC was originally considered across the two cohorts and undertaken by zipcode, n = 311 (Soloff et al., 2005). It was stratified geographically by state to cover both urban and rural communities with an average of 40 children per zipcode from the larger states and about 20 children per zipcode in the smaller states and territories were selected (Soloff et al., 2005). In the original sample of the K cohort at Wave 1, there were 4983 children and 3089 who remained to Wave 7 at age 16–17 years. In the sub-sample used in the current study there were 2723 participants.

### Measures

Measurement of study variables are parent report (Mohal et al., 2021).

#### Parental warmth

Parental warmth was assessed by primary caregiver reports from a six-item measure using a Likert-type scale (1–5), “never/almost never” to “always/almost always”. For example, “How often do you have warm and close times with this child?” A mean score was created with higher scores indicating increasing parental warmth for each wave (waves 1–6). The measure was drawn from the Child Rearing Questionnaire (Paterson & Sanson, 1999). LSAC data show internal consistency with alphas ranging from 0.92–0.95 across Waves 1–4 and construct validity from comparisons with other measures of family and parent characteristics (Zubrick et al., 2014).

#### Prosocial behavior

Prosocial behavior was measured using the five-item prosocial sub-scale of the Strengths and Difficulties Questionnaire (SDQ, Goodman et al., 1998) about the past 6 months using a Likert-type scale (0–2), “not true”, “somewhat true”, and “certainly true”. The prosocial scale score was collected at Wave 7 when the children were 16–17 years of age. The SDQ (parent report) is a well-established measure with previous assessment of the prosocial subscale’s showing adequate reliability (internal consistency and test-retest) (Goodman, 2001) including with Australian children (Hawes & Dadds, 2004). The Chronbach’s alpha in the current study is 0.68. As per the scoring recommendations (see Goodman et al., 1998) a total score was created and divided into three categories. In the original calculation of the bands they were termed, ‘normal’, ‘borderline’, and ‘abnormal’ however in this study the following terms were used instead, typical prosocial behavior (score = 6–10), near typical prosocial behavior (score = 5) and limited engagement in prosocial behavior (score = 0–4).

#### Demographics

Demographics were collected at Wave 1 including, child’s gender and age, caregiver gender, child is a First Nations Australian, language other than English spoken at home by the child, number of siblings, and socioeconomic position (continuous scale, described as calculated by using the parent’s income level, education, and occupation, see Baker et al., 2017).

### Data Analyses

#### Data selection and missing data

Analyses were conducted using the weighted data (longitudinal weights) from the Longitudinal Study of Australian Children to maintain representativeness (Usback et al., 2018). Weights reflect a modeled response propensity factor and stratum weight adjustment as well as capping of extreme weights (Usback et al., 2018). Part of the weighting process is designed to adjust for non-response by characteristics of families with differing likelihood of attrition. The longitudinal weights take into account the weight from the previous wave and adjust as necessary for the current wave (Usback et al., 2018).

At Wave 7, the non-response rate was 26%, the primary reasons were reported as refusal (45% of non-responders, e.g., “does not want to”, “too busy”) and non-contact (36%) (Bandara et al., 2020). The authors explored the characteristics of those who were non-responders with those who responded with differences on the variables (included in the current study) of, Aboriginal and Torres Strait Islander Australians and having more siblings (three or more compared with none) with both being more likely to be associated with those in the attrition group than the remaining group. However socio-economic area of residence (SEIFA), language spoken at home, and gender (child/parent) were tested but were not different between groups (Bandara et al., 2020).

To be included in the regression models participants needed to have a non-missing record for the outcome variable and all exposures included in that version of the model. The final multinomial model presented (Table 5) included 2723 participant records.

#### Descriptives

Analyses were conducted with Stata 17.0/SE version (StataCorp., 2021). Initial analyses undertaken included descriptives (means, standard deviations of study variables and correlations). The descriptives provided reflect those participants with data for the final hypothesis testing model. Supplementary data is provided showing descriptive data for those participants with data available at Wave 1 and those participants who are in the final hypothesis testing sample. This however does not reflect the weights that are applied after Wave 1.

Initial univartiate analyses were performed assessing the role of parental warmth measured at each wave as a predictor of prosocial behavior at age 16–17 years. Initial linear regression models fitted using the prosocial behavior outcome produced a skewed distribution of model residuals, therefore the strength of each wave of parental warmth on the outcome of prosocial behavior at 16–17 years was examined with multiple multinominal logistic regression models. An overall assessment was made for each explanatory variable using a Wald test. All analyses tested statistical significance with 95% confidence. A goodness of fit test (Hosmer-Lemeshow test) was used to assess how well the model fit the data for the multinomial logistic regressions (Fagerland & Hosmer, 2012).

#### Creating trajectories of parental warmth

To examine patterns of parental warmth over development and identify clusters within the longitudinal data, trajectory analyses were performed. A Group Based Trajectory Modeling (GBTM) approach (Jones & Nagin, 2013) that estimates discrete mixture models was chosen over a Generalized Mixed Models approach due to the anticipated simpler structure and interpretability likely to result from the GBTM method (Nguena Nguefack et al., 2020*)*. Initially two to six trajectory groups were examined and statistical fit and parsimony assessed. To select the appropriate number of groups the BIC value was examined. The model with the highest (least negative) BIC value indicates better fit (Nagin, 2005). The BIC was used given findings which suggested it provides the best indicator of number of classes of the information criterions (Nylund et al., 2007). In addition to the BIC, the final selected trajectories also reflect the most parsimonious model that best describes distinguishing and interpretable patterns (Nagin & Odgers, 2010).

The shape of each trajectory group were determined. Results suggested that a 1, 2, 2 order fit the data best, a linear, quadratic, quadratic pattern. Posterior probabilities for each trajectory group were assessed as at least 0.90 as an indication of adequate fit. An acceptable model fit is a minimum average posterior probability of 0.70 for all group trajectories (Nagin & Odgers, 2010).

Three trajectory groups were modeled with one linear and two quadratic terms, with parental warmth as the longitudinal variable from a minimum of three waves (waves 1-76, 87.28% of the original sample) and age as the time variable (ages 4–5, 6–7, 8–9, 10–11, 12–13, and 14–15 years). Groups reflected likely trajectories named, a consistent high parental warmth, a slight decline and a declining trajectory. While a four group approach fit the data (based on the BIC value), it was less parsimonious and the additional trajectory identified provided a very similar shape as the slight decline group.

#### Hypothesis testing

To explore the role of alignment with the newly created trajectories on prosocial behavior an adjusted multinomial logistic regression model was used. The outcome was prosocial behavior in three categories of, typical, near typical, and limited engagement in prosocial behavior (following recommended categorization, see Goodman et al., 1998). The main predictor was trajectory of parental warmth and included categories of, consistently high parental warmth, slight decline, and declining warmth. Of note, these are latent categories (not observed) as derived from the initial probability estimates and reflect individuals grouped based on a similar trajectory of parental warmth rather than distinct entities (Nagin & Odgers, 2010). Covariates included child’s gender, First Nations Australian, speaking a language other than English at home, number of siblings, caregiver gender, and socioeconomic position (SEP). A multinomial logistic regression was also performed without the longitudinal weightings (Usback et al., 2018) as a sensitivity analysis but findings were consistent.

The Hosmer Lemeshow goodness of fit test was used again to assess how well the model fit the data. The default number of groups (n = 10) and a p-value of >0.05 continued to serve as a guideline for a model to be considered to have a good fit. Given there was not the aim to compare models and all covariates are included in the regression only the single goodness of fit test was included. A linear regression was also performed with a continuous outcome variable (prosocial behavior) and predictors were largely consistent; with the exception of socio-economic position and number of siblings now significant predictors (see Supplementary File). The multinomial logistic model is presented as the primary findings given the violation of assumptions of the linear model (skew of the residuals in the outcome, prosocial behavior), and for interpretability based on the original scoring categories proposed (Goodman et al., 1998).

## Results

### Sample Overview

Few children spoke a language other than English at home (n = 241, 8.9%), there was an even gender split between female (n = 1339, 49.2%) and male (n = 1384, 50.8%) children and 1.9% (n = 52) were First Nations Australians. Primarily the gender of the primary caregiver was female (n = 2644, 97.1%) compared with male (n = 79, 2.9%). See Table 1 for correlations among the main variables.

**Table 1:** Correlations of predictor variables

	1	2	3	4	5	6	7	8	9
1 Warmth trajectory - high	1								
2 Warmth trajectory - slight decline	−0.64***	1							
3 Warmth trajectory - decline	−0.32***	−0.52***	1						
4 Child’s gender is male	−0.02	−0.02	0.05*	1					
5 Parent’s gender is male	−0.04*	0.03	0.01	0.01	1				
6 Language other than English	−0.02	−0.01	0.03	−0.01	0.04*	1			
7 First Nations Australian	−0.02	0.01	0.01	−0.01	−0.01	−0.03	1		
8 Socioeconomic position	−0.01	−0.01	0.01	0.01	−0.02	0.03	−0.07***	1	
9 Siblings	−0.07***	−0.01	0.09***	−0.02	−0.02	−0.03	0.02	−0.07***	1

*N* = 2723. Reference group for the trajectories are not membership in the trajectory. Reference groups for gender are female

*p < 0.05, **p < 0.01, ***p < 0.001

### Parental Warmth Across Childhood

There is a general trend of decreased parental warmth with age as shown in Table 2.

**Table 2:** Descriptive statistics of parental warmth by age

Age in years (study wave)	Parental warmth score	
	*Mean*	*SD*
4–5 (wave 1), n = 2989	4.43	0.45
6–7 (wave 2), n = 2857	4.43	0.48
8–9 (wave 3), n = 2672	4.31	0.55
10–11 (wave 4), n = 2923	4.27	0.59
12–13 (wave 5), n = 2891	4.16	0.63
14–15 (wave 6), n = 2823	4.03	0.69

**Fig. 1:**
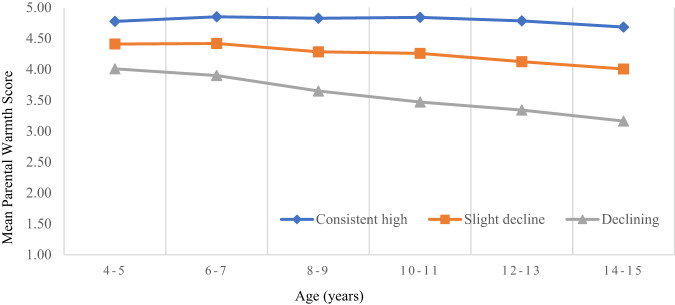
*Mean Parental Warmth Score at Each Age for the Trajectories of Parental Warmth. Note*. *N* = 4348. Errors are consistently small. Consistently high parental warmth standard errors range, 0.007–0.012; slight decline, 0.008–0.011; declining, 0.016–0.019

Three main trajectory groups of parental warmth across childhood were created and shown in Fig. 1. These classes suggest that on balance there are children who can be described as consistently high on parental warmth (n = 834, 28.7%), a slight decline in parental warmth (n = 1551, 51.4%), and a declining parental warmth (n = 603, 19.8%).

### Parental Warmth and Prosocial Behavior

Most children meet the criteria for typical prosocial behavior (n = 2686, 90.01%). Almost all children who fall in the consistently high parental warmth class also display typical prosocial behavior (n = 797, 95.91%). There are very few children who display limited prosocial behavior and can be described as consistently high on parental warmth (n = 16, 1.92%). Table 3 shows the percent of children in each of the parental warmth trajectories across the outcome variable of categories of prosocial behavior at age 16–17 years.

**Table 3:** Number and proportion of adolescents in the parental warmth trajectory groups and each category of prosocial behavior (*N* = 2988)

Prosocial behavior at 16–17 years	Parental warmth trajectories			
	Consistently high warmth	Slight decline in warmth	Declining warmth	Total (all trajectories)
	n (%)	n (%)	n (%)	n (%)
Typical	800 (95.92)	1420 (91.55)	471 (78.11)	2691 (90.06)
Near typical	18 (2.16)	75 (4.84)	77 (12.77)	170 (5.69)
Limited engagement	16 (1.92)	56 (3.61)	55 (9.12)	127 (4.25)

Greater parental warmth was associated with likelihood of typical prosocial behavior at each age of assessment compared with near typical or limited prosocial behavior (see Table 4, all models had good fit). The finding that increased parental warmth lowers the likelihood of near typical or limited prosocial behavior is consistently observed at each age point. For example, this is evident from age 4-5 years (RRR = 0.47; 95%CI 0.35–0.63, *p* < 0.001), for a 1-unit increase in parental warmth it is associated with a lower likelihood of near typical prosocial behavior compared with typical prosocial behavior.

**Table 4:** Relative risk ratio (RRR) of prosocial behavior at 16–17 years of age from parental warmth measured across development from multinomal logistic regression analyses

Prosocial behavior category	RRR^a^	95% CI	Model Goodness of Fit: χ^2^, *p*
Model 1, parental warmth at wave 1 (4–5 years), n = 2989			12.24, 0.728
Near typical	0.47	0.35, 0.63	
Limited	0.46	0.33, 0.64	
Model 2, parental warmth at wave 2 (6–7 years), n = 2857			20.75, 0.188
Near typical	0.45	0.34, 0.60	
Limited	0.41	0.29, 0.58	
Model 3, parental warmth at wave 3 (8–9 years), n = 2672			11.73, 0.762
Near typical	0.40	0.31, 0.51	
Limited	0.46	0.34, 0.63	
Model 4, parental warmth at wave 4 (10–11 years), n = 2923			23.83, 0.093
Near typical	0.40	0.31, 0.50	
Limited	0.38	0.29, 0.50	
Model 5, parental warmth at wave 5 (12–13 years), n = 2891			18.31, 0.306
Near typical	0.41	0.33, 0.52	
Limited	0.36	0.28, 0.47	
Model 6, parental warmth at wave 6 (14–15 years), n = 2823			9.33, 0.899
Near typical	0.40	0.32, 0.51	
Limited	0.32	0.25, 0.42	

All RRRs are *p* < 0.001

^a^Reference category = Typical prosocial behavior. Models adjusted for age of study child at each individual wave, and Wave 1: gender of the study child, gender of the main caregiver at, number of siblings, social economic position, languages other than English spoken at home by the study child, and First Nations Australian

### Predicting Prosocial Behavior from Parental Warmth Trajectories

Again parental warmth predicted prosocial behavior and analyses showed those who likely fit a trajectory of consistently high parental warmth also have the positive outcome (see Table 5). The model had good fit, χ2 = 14.85, df = 16, p = 0.54, and controlled for covariates (with only child’s gender contributing to the model).

**Table 5:** Summary of the adjusted multinomial logistic regression on prosocial behavior at 16–17 years

	Near prosocial behavior		Limited prosocial behavior	
Explanatory Variable	RRR	95% CI	RRR	95% CI
Parental warmth trajectories (^a^Consistently high)				
Slight declines in warmth	2.20	1.15, 4.24*	2.29	1.15, 4.53*
Declining warmth	8.53	4.56, 15.97***	6.62	3.38, 12.94***
Child’s gender is male (^a^female)	1.78	1.19, 2.66**	1.90	1.18, 3.05**
Language other than English (^a^no)	1.43	0.77, 2.66	1.22	0.62, 2.40
First Nations Australian (^a^no)	0.94	0.23, 3.77	1.29	0.35, 4.70
Caregiver gender is male (^a^female)	1.68	0.63, 4.46	1.57	0.50, 4.95
Number of siblings	0.96	0.77, 1.20	0.98	0.75, 1.28
Socioeconomic position	0.85	0.70, 1.03	0.89	0.69, 1.14

*N* = 2723

Model fit: *χ*2 = 14.845, df = 16, *p* = 0.536

*p < 0.05, **p < 0.01, ***p < 0.001

^a^Baseline categories. Reference group for prosocial behavior groups is typical prosocial behavior other reference groups noted in text

Children in the group described as slight declines of parental warmth from 4–5 years through to 14–15 years were more than two times likely to be in the near typical versus typical prosocial behavior group at 16–17 years compared to those who fall into the class of those who might experience consistently high levels of parental warmth across their childhood years (RRR = 2.20, 95%CI; 1.15–4.24; *p* = 0.018). Further, children in the class of declining parental warmth were 8.53 times more likely to be near typical in prosocial behavior at 16–17 years compared to those in the latent trajectory of consistently high levels of parental warmth (RRR = 8.53, 95%CI; 4.56–15.97, *p* < 0.001). Those in the latent trajectory of declining parental warmth over time were more than six times more likely to have limited prosocial behavior at age 16–17 compared to those in the consistently high levels of parental warmth trajectory (RRR = 6.62, 95%CI; 3.38–12.94, *p* < 0.001).

## Discussion

Prosocial behavior is a key social skill and is associated with numerous other positive outcomes for an adolescent (Memmott-Elison et al., 2020). Previous studies had shown parental warmth is associated with prosocial behavior (Padilla-Walker et al., 2016) however had not looked at the relationship when considering parental warmth to be heterogenous and thus had not used a trajectory approach across development to explain prosocial behavior. In seeking to understand the experience of parental warmth with latent developmental trajectories, it provides a person-centered (rather than variable-centered) approach providing qualitatively and descriptively distinct trajectories of parental warmth. This study examined the role of trajectories of parental warmth through childhood and adolescence on prosocial behavior at age 16–17 years old. There were three trajectories and these predicted likelihood of later prosocial behavior.

Findings suggest that for most, parental warmth declines particularly around early adolescence. The trajectory analyses of parental warmth suggests three distinct groups, including a group who can be described as, consistently high on parental warmth, those with some minor decline (the majority), and those with some more consistent decline over time. As found with mother-son relationships, overall parental warmth may be heterogenous (unobserved) through child and adolescent development and there are subgroups (Trentacosta et al., 2011). It supports a distinction in parent-child relationships where there may not always be a consistently high experience across the school years.

At the beginning of this developmental period, children are entering school and by mid-adolescence they are embarking on markedly different social interactions of increasing independence. The typical role of parents is perhaps changing where in childhood there is the warmth and scaffolding to promote positive social interactions that is reduced through adolescence as it aligns with increasing independence (e.g., Maccoby & Martin, 1983).

The findings are aligned with other work that has examined trajectories in other parent-child relationship factors, for example there were heterogenous trajectories in both support and conflict. These trajectories were associated with differences in socio-emotional experiences, including romantic relationship quality (Seiffge-Krenke et al., 2010) and anxiety (Kim et al., 2015). Understanding the trajectories may ultimately help inform tailored interventions and more optimal support from limited resources (Zheng et al., 2017). There is potential that the heterogeneity explains some of the variation in some study results where there are mixed findings of the role of parental warmth in its association with adolescent behavior. For example, where there may only be indirect or limited role in parental warmth explaining likelihood of externalizing behaviors (Padilla-Walker et al., 2016).

Findings suggest potential value in maintaining parental warmth across development, at least when it comes to prosocial behavior. While the group described as consistent parental warmth was less common, compared with some slight gradual decline in warmth, attempts to maintain or provide strategies for parents to continue warmth through adolescent years may have benefits. Parenting programs and resources that improve warmth may be valuable, where warmth typically declines at around puberty (Lansford et al., 2021). Future parenting programs and resources might benefit from recognizing parents’ strengths through childhood and build on those earlier skills as well as strategies to provide warmth in the context of adolescence and key development tasks around gaining independence, particularly in the context of prosocial behavior.

The trajectories were differently associated with reported prosocial behavior. Further, at each assessment point in child or adolescent development there was an association between parental warmth and the later prosocial behavior. As children got older, higher levels of parental warmth provided an even greater reduction of risk. Despite the strength of the more proximal relationship, early experiences also predict later prosocial behavior and thus there are potentially multiple points at which parents’ behavior may be important and potentially multiple opportunities to provide support and resources for parents across their child’s development.

There were several covariates included in analyses, with only child’s gender playing a role in predicting prosocial behavior (when other variables were considered in the model). Such a finding is consistent with previous research that being female is predictive of greater prosocial behavior (Malonda et al., 2019). Future research, where possible should expand the considerations of other aspects of a child’s ecology beyond basic demographics, including, for example, gender identity more broadly and the wider context in which children live and play.

The research should be considered within the context of strengths and limitations. Both parental warmth and adolescent prosocial behavior were measured through the responses received from the parent (main caregiver), which lacks the voice of the adolescents. While it potentially provides detail about the provision of warmth and the observation in some situations of prosocial behavior, there is variation in rates of prosocial behavior when assessed by different individuals (Padilla-Walker et al., 2016) and when using a multi-dimensional conceptualization of prosocial behavior (Carlo & Randall, 2002). Related, the responses are solely from the one reporter (parent) and as such there is potential for correlations to be artificially inflated (mono-method reporter bias). There are also differences in prosocial behavior depending on the intended recipient of prosocial behavior (Padilla-Walker et al., 2018), as such a more detailed understanding and assessment of prosocial behavior and the relationship with different trajectories of warmth is warranted.

Interestingly, the majority (around half) are reflected in a group showing a decline in parental warmth over time, while this group showed less prosocial behavior (than those with consistent warmth) it might be that parental warmth changes through development and this is not well captured when using the same measurement tool of warmth over time. Future research might consider more conceptually how parental warmth evolves and as it relates to adolescent socio-emotional skills more broadly (including a multi-dimensional concept of prosocial behavior). The study focused on testing the theory that the early social experience (parental warmth) would explain later prosocial behavior but it does not consider the trajectories of prosocial behavior and the way in which early engagement in prosocial behavior might impact the trajectory of parental warmth. Further, while key demographic factors were statistically controlled, the focus was on explaining prosocial behavior later in adolescence. Future research would benefit from understanding the factors that promote parental warmth in the way that it explains prosocial behavior, including earlier child behaviors and contextual factors.

## Conclusion

Prosocial behavior is associated with a wide array of positive outcomes. While earlier experiences of parental warmth have been associated with such behavior, the research to date has largely not considered warmth to be described heterogeneously across development. The current study showed unique latent trajectories of parental warmth through school-aged development where consistent warmth was associated with a reduction in risk from both near-typical and limited prosocial behavior by 16–17 years of age. Considering parental warmth can be modifiable, the findings suggest a potential focus for future efforts to increase adolescents’ prosocial behaviors. Such behaviors that are associated with a positive impact on the success of the child development in academia, health, and their interpersonal and community interactions.

### Supplementary information

Supplementary Information

## Data Availability

The data that support the findings of this study are available from the Longitudinal Study of Australian Children but restrictions apply to the availability of these data, which were used under license for the current study, and so are not publicly available. However, data are available with permission from the Longitudinal Study of Australian Children.
