# Supplementary material for: Trajectories of Parental Warmth and the Role They Play in Explaining Adolescent Prosocial Behavior
Source: J Youth Adolesc. 2023 Oct 21;53(3):526–36. doi: 10.1007/s10964-023-01887-3 (PMC10838220; doi:10.1007/s10964-023-01887-3)
Supplement: Supplementary file 1 — Supplementary Information [file 10964_2023_1887_MOESM1_ESM.docx]

**Supplementary File 1- Comparison of Demographic Data**

**Table S1.** Categorical Covariates at Wave 1: Comparison of Demographics With All Available Data and Data Selected With Parental Warmth and Prosocial Behavior.

|  | Wave 1 - all (N=4983) | | Wave 1 – Parental warmth trajectory & prosocial behavior (N=2723) | |
| --- | --- | --- | --- | --- |
| Variable | n | % | n | % |
| **Gender of study child** | | | | |
| Female | 2447 | 49.1 | 1339 | 49.2 |
| Male | 2536 | 50.9 | 1384 | 50.8 |
| **Gender of main caregiver** | | | | |
| Female | 4839 | 97.1 | 2644 | 97.1 |
| Male | 144 | 2.9 | 79 | 2.9 |
| **Languages spoken other than English at home by the study child** | | | | |
| No | 4359 | 87.5 | 2482 | 91.1 |
| Yes | 624 | 12.5 | 241 | 8.9 |
| **Indigenous Australian** | | | | |
| No | 4794 | 96.2 | 2671 | 98.1 |
| Yes | 187 | 3.8 | 52 | 1.9 |
| **Number of siblings**  (Mean, SD; Min-Max: 0-11) | 1.49 | 1.1 | 1.4 | .9 |
| **SEP** (Mean, SD) | 0.00 | 1.0 | .2 | 1.0 |

**Supplementary File 2 – Linear Regression Analysis**

Below are the linear regression analyses whereby the outcome variable of prosocial behavior is scored as a continuous variable. Of note, these are provided as a supplementary file as the assumption of normality of residuals is violated for the outcome variable.

**Table S2**. Linear Regression With the Outcome of Prosocial Behavior at 16 Years of Age.

| Explanatory Variable | Coefficient | S.E. | t | 95% CI |
| --- | --- | --- | --- | --- |
| Parental warmth trajectories (^a^Consistently high) | | | | |
| Slight declines in warmth | -.59 | .08 | -7.63*** | -.74, -.44 |
| Declining warmth | -1.47 | .11 | -13.92*** | -1.68, -1.26 |
| Child’s gender is male (^a^female) | -.48 | .07 | -6.46*** | -.63, -.34 |
| Language other than English (^a^No) | -.03 | .13 | -.20 | -.29, .23 |
| First Nations Australian (^a^No) | .02 | .31 | .06 | -.58, .62 |
| Caregiver gender is male (^a^female) | -.27 | .29 | -.91 | -.84, .31 |
| Number of siblings | .09 | .04 | 2.08* | .00, .18 |
| Socioeconomic position | .17 | .04 | 4.06*** | .09, .25 |

^a^Reference group for prosocial behavior groups is typical prosocial behavior other reference groups noted in text.

N=2723. *p<.05, **p<.01, ***p<.001.

Model fit: F(8,260) = 36.26, p<.001; Adj R^2^ = .11.
